# Examination of Physical Activity Patterns of Children, Reliability and Structural Validity Testing of the Hungarian Version of the PAQ-C Questionnaire

**DOI:** 10.3390/children10091547

**Published:** 2023-09-14

**Authors:** Alexandra Makai, Viktória Prémusz, Olívia Dózsa-Juhász, Kitti Fodor-Mazzag, Csaba Melczer, Pongrác Ács

**Affiliations:** 1 Institute of Physiotherapy and Sport Sciences, Faculty of Health Sciences, University of Pécs, H-7621 Pecs, Hungary; premusz.viktoria@pte.hu (V.P.); pongrac.acs@etk.pte.hu (P.Á.); 2 Physical Activity Research Group, Szentágothai Research Centre, University of Pécs, H-7621 Pecs, Hungary

**Keywords:** active living, student, psychometrics, confirmatory factor analysis

## Abstract

Introduction: Several studies report on the importance of physical activity (PA) in childhood, which influences attitudes towards health in adulthood. For monitoring PA, trustworthy measurement tools are needed. The study aimed to adapt the Physical Activity Questionnaire for Children (PAQ-C) to the Hungarian language and assess its validity, reliability, and factor structure. Methods: A total of 620 children (the average age was 10.62 (SD 2.36)) participated in the cross-sectional study. To assess physical activity, the PAQ-C questionnaire was used. The collected data were analysed using IBM SPSS version 28.0 and IBM SPSS AMOS 29.0 software. Results: The internal consistency was acceptable (alpha = 0.729) and the test-retest reliability showed acceptable agreement (ICC = 0.772). The confirmatory factor favoured a one-factor structure of the questionnaire. The average PAQ-C score for girls was 2.87 (SD 1.07), and for boys it was 3.00 (SD 1.05), which showed a significant difference (*p* = 0.005). Discussion: Based on our findings, our study tested the validity and reliability of the one-factor PAQ-C questionnaire, a valid and reliable measurement tool to test the physical activity patterns of primary school children in a Hungarian sample. Further research is needed to develop physical activity monitoring of Hungarian children.

## 1. Introduction

Physical inactivity is associated with a significant risk of cardiovascular and chronic diseases such as obesity, hypertension, diabetes mellitus, and musculoskeletal conditions such as osteoporosis, osteoarthritis, and mental health [1]. According to the World Health Organization (WHO), in 2003, about 60% of the world’s population lead inactive lifestyles, the consequences of which placed a heavy burden on healthcare systems worldwide [2]. 

Several research findings report on the importance of physical activity in childhood, which influences attitudes towards health in adulthood [3,4,5]. Physical activity (PA) in youth has several benefits, including cardiorespiratory fitness, normal muscle strength, and mental status, and reduces the risk of obesity and certain metabolic diseases [6]. In addition, physical activity at a young age has been shown to have an impact on the development of cognitive function and, thus, academic performance [7]. 

The recommendations for physical activity levels in children vary slightly between the WHO and European and American guidelines. The WHO recommends that children and adolescents aged 5–17 engage in at least 60 min of moderate-to-vigorous-intensity physical activity daily. This can include active play, sports, recreational activities, and structured exercise. Additionally, the WHO suggests incorporating activities that strengthen muscles and bones at least three times a week. The European guidelines align closely with the WHO recommendations. They recommend that children and adolescents aged 5–17 get a minimum of 60 min of moderate-to-vigorous-intensity physical activity daily. The guidelines emphasize various activities, including aerobic exercise, muscle-strengthening activities, and activities promoting bone health. However, the American guidelines provide more specific recommendations based on age groups; for children and adolescents (6–17 years), the recommendation is at least one hour of moderate-to-vigorous-intensity physical activity daily. This should include a combination of aerobic exercises and muscle-strengthening and bone-strengthening activities [8,9].

Several studies have shown that a significant proportion of children in Europe do not meet the WHO’s recommended physical activity levels or are inactive. The European Union-funded project called the Healthy Lifestyle in Europe by Nutrition in Adolescence Study (HELENA) examined adolescents’ physical activity levels across several European countries. The study found that many adolescents fell short of meeting the recommended 60 min of daily moderate-to-vigorous-intensity physical activity and spent more than 9 h/day sedentary [10].

Similarly, the Global Matrix 3.0, which assesses physical activity levels and behaviours in children and youth across multiple countries, including several European nations, reported low levels of physical activity among children. Furthermore, joint monitoring tools are needed for better comparison and monitoring [11]. 

The Health Behaviour in School-aged Children (HBSC) international survey conducted in 2017/18 provided valuable insights into the health behaviours of school-aged children across various countries. The HBSC survey is conducted every four years. It involves gathering data on multiple aspects of young people’s health and well-being, including physical activity, sedentary behaviours, nutrition, mental health, and social relationships. The survey typically covers various countries, providing a comparative analysis of health behaviours among school-aged children. The study found that higher levels of social media use were associated with an increased likelihood of experiencing mental health problems among school-aged children. Specifically, excessive social media use was linked to higher levels of psychological distress, depressive symptoms, and poor self-esteem [12]. 

Adequate tools are needed to monitor physical activity levels accurately, but this can be challenging in many cases given that physical activity is a complex, multidimensional human behaviour [13,14]. There are many objective measures of physical activity levels, but these have yet to be shown to be applicable to large study samples [15]. For all these reasons, self-completion questionnaires and physical activity questionnaires (PAQs) are becoming increasingly common, as they are inexpensive, multisided, and easy-to-use measurement tools. However, PAQs can present more limitations in understanding the questions of the measurement tools, and calculating the time spent physically active within one week could be difficult for children and could mean an overestimation of the physical activity level [16,17].

There are several questionnaires for assessing physical activity in children, such as the Health Behaviour in School-Aged Children Questionnaire and the Youth Activity Profile [18,19,20,21]. However, the most frequently used PAQ for children is the Physical Activity Questionnaire for Older Children (PAQ-C), ranked as one of the few self-report instruments with acceptable validity, reliability, and practicality for assessing physical activity levels among children. The PAQ-C questionnaire was developed in Canada in 1997 by Kowalski et al. for use in the ALPHA study. Therefore, it can provide a high standard for comparisons beyond the European standards [22,23,24]. The questionnaire measures the weekly average physical activity patterns of the children aged 8–14 years during school time (except summer holidays) about sports activities, leisure time activities, physical education classes, and during school time. 

In the previous studies, the PAQ-C questionnaire showed acceptable validity and reliability. The measurement tool is adapted and validated into more languages, such as German, Dutch, Greek, Italian, Croatian, Chinese, Czech, Spanish, Japanese, Turkish, and Saudi Arabian. The results of these studies investigated the internal consistency of the questionnaire, and more studies assessed the test-retest reliability. The convergent validity of the PAQ-C compared with accelerometer (generally measured by Actigraph GT3X) results showed moderate correlation and acceptable reliability between the PAQ-C scores and vigorous, moderate, or moderate to vigorous physical activity (MVPA) weekly minutes or metabolic equivalent (MET)/minutes or daily steps. Furthermore, the measurement tool’s structural validity using confirmatory factor analysis (CFA) was examined in Saudi Arabian and Turkish validation studies, where the first study confirmed the one-factor structure and the second confirmed the two-factor structure of the PAQ-C [25,26,27,28,29,30,31,32,33]. 

Before the current study, there was no validated subjective measurement to examine the PA patterns of children in Hungary. The HBSC study also measured Hungarian children’s physical activity patterns, and they found that less than 20% of them met the recommended level of PA [34]. However, the last decade has seen significant changes in monitoring children’s activity in Hungary. The developed National Standardized Student Fitness Test (NETFIT) system, in four fitness profiles, examines nine measurements to characterize students’ physical fitness, strength, flexibility, and body composition. NETFIT is used by more than 3700 schools, 800,000 children, and 13,000 teachers. Current research supports the existing monitoring of children’s physical fitness with the tracking of physical activity patterns [35,36]. 

This study aimed to adapt the PAQ-C to the Hungarian language for assessing physical activity among children and to determine the measurement tool’s psychometric properties by testing its validity, reliability, and factor structure. 

## 2. Materials and Methods

A cross-sectional survey was conducted with the participation of primary school students. The data were collected from January 2020 to March 2023 in the south-Danubian region of Hungary. 

The applied sampling procedure was convenient sampling. The calculation of the minimum sample size was based on previous studies [30,37], a ratio of ten participants per item for CFA [33], and a recommendation of 300–500 participants [32]. We recruited children from 7 public primary schools. Male and female, 7–14-year-old school-going children were recruited. Regarding exclusion criteria, the sample could not include children with disabilities regarding physical activity patterns. 

**Figure 1:**
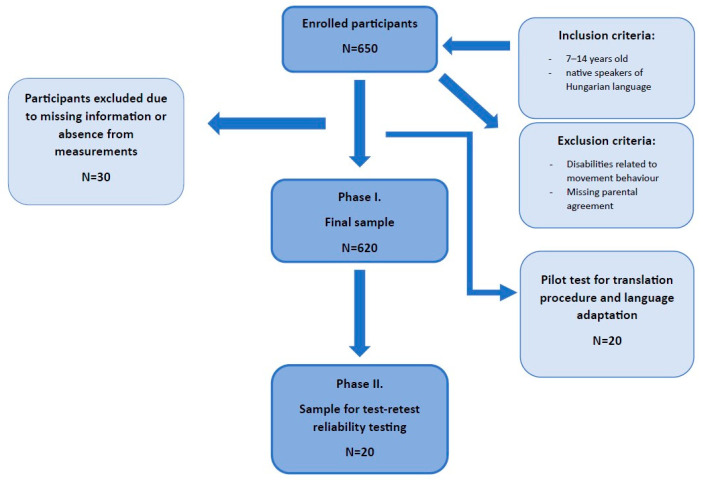
Flow chart of sample and recruitment.

In total, 650 children were recruited into the study, and the final sample consisted of 620 participants involved in the final statistical analysis in Phase 1. Thirty participants were excluded because of age requirements, sick leave, or missing data. From the recruited sample, 20 children were involved in the test-retest measurement in Phase 2 of the research, where all participants filled out the questionnaire again after 7 days and were included in the final analysis. The sample and recruitment procedure were summarised in a flow chart (Figure 1). 

### 2.1. Ethical Approval and Consent to Participate

Participation in the research was voluntary. All participants and their parents were informed about the details of our study on a written informed consent form. Children were able to enter the study after written parental consent.

The study was approved by the ETT TUKEB, Hungary (15117-9/2018/EÜIG). All the methods used were carried out under relevant guidelines (Beaton’s and COnsensus-based Standards for the selection of health Measurement Instruments (COSMIN) guidelines and regulations) [38,39]. The data were processed anonymously and confidentially based on the Data Protection Act of Hungary. The research was conducted by the principles of the Declaration of Helsinki. 

#### 2.1.1. Assessment Tools

##### PAQ-C

The PAQ-C questionnaire is a ten-item, self-administered, 7-day recall questionnaire for children aged 8–14. The total score of the measurement tool is the mean score of the measured scales ranked between 1 and 5, where a higher value means a higher level of moderate and vigorous intensity physical activity based on the first 9 questions of the PAQ-C and 1 question about the health status of children. The first question contained the different activities and ratings, how often they participated in them, and children’s rating between 1 and 5, and an average score was calculated from the results of the first question. The following 8 questions were rated between 1 and 5, and the final total score of the questionnaire was the average score of the 9 questions of PAQ-C, where a higher value means a higher level of physical activity [23]. 

##### Demographic Questions and Body Composition

The study obtained the gender, age, parent’s education, and general health status of the participants. Trained physiotherapists measured the body composition of the students using InBody_770_ (InBodyUSA, Cerritos, CA, USA); their weight was measured in kg and their height in cm. Their body mass index (BMI, kg/m^2^), body fat, body fat %, and skeletal muscle index were calculated. 

### 2.2. Adaptation and Validation Procedure

The Hungarian version of the PAQ-C questionnaire was obtained from the original author of the questionnaire via email [23]. First, the PAQ-C questionnaire was adapted to the Hungarian language using Beaton’s guidelines. After the pilot testing (N = 20) of the new measurement tool, the psychometric properties of the questionnaires were measured. The language adaptation of the questionnaire was developed by independent professional translators from the field of health science and education, and then the translators prepared the back translation of that. Based on the developed versions of the PAQ-C with the agreement of the translators, the research committee finalized the final version of the questionnaire. The expert committee consisted of experts in physical activity monitoring, physical education (PE) teachers, physiotherapists, English and Hungarian language teachers, and statisticians. 

The activity list of the questionnaire was modified; a few activities were deleted (street hockey), and cross-country skiing was limited to skiing/snowboarding. In the pilot test phase of the validation procedure, a group of students (aged 8–14 years, N = 20) answered and filled out the PAQ-C based on the results of the pilot testing of the measurement tool. 

The Flesch reading index was applied to test the readability of the PAQ-C. The scores range from 0 to 100, and a value of more than 60 means participants can readily understand the tool [40]. Based on the results of the reading score, the PAQ-C questionnaire was considered a straightforward reading measurement tool (Flesch Reading Ease Score = 97.8). 

To examine the test-retest reliability of the measurement tool, one primary school was selected, recruiting 20 participants from more school classes in Phase 2 of our study. The participants filled in the questionnaire two times, and after 7 days of the first measurements, they repeated the filling out of the PAQ-C questionnaire. 

### 2.3. Statistical Analysis

For descriptive statistics, we utilized various statistical calculations, including minimum, maximum, mean (± standard deviation (SD)), and median (interquartile range) to provide characterization. The descriptive analysis of measured items was carried out separately for the total sample and for girls and boys. The CFA was used to test the factor structure and structural validity of the PAQ-C questionnaire. The applied fit indexes were the chi-square test, chi-square/degree of freedom (df) ratio, comparative fit index (CFI), Tucker Lewis index (TLI) and Root Mean Square Error of Approximation (RMSEA) indexes. The criteria for the tests were the following based on the previous research and recommendations: a χ^2^ and χ^2^/df ratio less than 3 indicated a good fit, RMSEA < 0.05 excellent, between 0.05 and 0.08 acceptable, TLI and CLI above 0.95 excellent, above 0.90 acceptable [31,41]. 

To measure the internal consistency of the questionnaire, Cronbach’s alpha was calculated. The value was consisted acceptable >0.70 [42,43].

The absolute agreement was calculated to examine the test-retest reliability intraclass correlation coefficient (ICC) with 95% confidence interval (95%CI) with a two-way mixed effect model. The ICC results were considered excellent above 0.80 [44,45]. 

The concurrent validity of the PAQ-C and body composition results was assessed using Spearman’s rank correlation coefficients, which were interpreted as weak if <0.03; 0.30–0.05 moderate; 0.50–0.75 good correlation; >0.75 excellent [32,46].

The discriminant validity of the questionnaire between male and female subgroups was tested using the Mann–Whitney U test to test the difference between the total score of PAQ-C by gender [47,48]. 

The statistical analysis of the study involved using IBM SPSS version 28.0 and IBM SPSS AMOS 29.0 (SPSS Inc., Chicago, IL, USA) software. The significance level was set at *p* < 0.05.

## 3. Results

The average age of the respondents was 10.62 (SD 2.36) years. The average BMI was 18.46 (SD 3.71) kg/m^2^. A total of 45.48% of the students were girls and 54.52% were boys. The parent’s education level was relatively high: 60.12% of mothers and 50.81% of the fathers had college or university degrees. In addition, 84.19% of the children lived in cities. 

The main characteristics of the sample are summarized in Table 1.

In our study, we also measured the body composition of the sample; Table 2 presents, separately for male and female participants and for the total sample, the results of that, where significant differences were found in body fat (*p* < 0.001), skeletal muscle index (*p* = 0.005), and body fat % (*p* < 0.001) by gender.

### 3.1. Physical Activity Patterns

The average PAQ-C score was 3.00 (SD 1.05). The descriptive analysis of the PAQ-C questionnaire’s items by male and female participants and for the total sample is presented in Table 3. 

### 3.2. Internal Consistency and Test-Retest Reliability

The two measured reliability scores’ results showed good test-retest reliability of PAQ-C. The Cronbach alpha value of the total sample was at an acceptable level, 0.729. The test-retest reliability analysis showed excellent reliability, and the total PAQ-C score’s ICC value was 0.772 (95% CI 0.373–0.841). The two measured reliability scores’ results showed good test-retest reliability for the Hungarian version of the PAQ-C (Table 4).

### 3.3. Structural Validity of PAQ-C

To measure the structural validity of the questionnaire, a CFA was conducted. 

The CFA conducted on the Hungarian sample showed a good fit. 

The examined model’s specifications for the sample resulted in high and significant χ^2^ values due to the relatively large sample size. The one-factor model was justified after comparing the goodness-of-fit indicators for the models. 

In Model 1, based on the previous literature, a bifactorial structure was performed, which showed a partly acceptable factor structure. 

In Model 2, an unifactorial factor structure was performed, which showed better fit than Model 1, but all fit indexes. All fit indexes were acceptable and excellent in the structural validity testing except the chi-square test in the final model (Model 3), where the χ^2^ = 57.79 (*p* < 0.001), df = 25, χ^2^/df = 2.311, GFI = 0.98, TLI = 0.961, CFI = 0.973, and RMSEA = 0.046. Furthermore, in the unifactorial model, question 5 and 6 and 6 and 7 were signed as covariates (Table 5).

### 3.4. Concurrent Validity of PAQ-C Questionnaire

The concurrent validity of the PAQ-C was measured using Spearman’s rank correlation (Spearman’s r) between the PAQ-C total score and body composition indexes. A significant weak correlation was found between body fat, body fat %, and sceletal muscle index and the PAQ-C scores in the total sample, and among male participants, there was no significant association between body fat, body fat %, and PAQ-C score among female participants (Table 6).

### 3.5. Discriminant Validity

The Hungarian version of the PAQ-C questionnaire showed a significant difference between girls and boys (*p* = 0.005). The average PAQ-C score for girls was 2.87 (SD 1.07), and for boys it was 3.00 (SD 1.05).

## 4. Discussion

The current study provided the first valid measurement tool to examine the physical activity patterns of Hungarian children. The examination of the psychometric properties showed a good-fit factor structure for the unifactorial PAQ-C questionnaire and acceptable internal consistency and test-retest reliability. 

The translation procedure was performed based on the guidelines and previous studies. The questionnaire was successfully adapted to the Hungarian language with minor modifications to the original questionnaire, only in the first question’s activity list.

The descriptive analysis of the questionnaire showed a 3.00 (SD 1.05) mean value for the Hungarian total sample. The relatively high mean values compared with the other studies were due to the increased physical education mean value of the children, which was 4.17 (SD 0.99). The Chinese (4.04 SD 0.80) and Turkish (4.52 SD 1.00) research found similar average values, like the current study, higher than 4 points [28,30]

The second-highest activity score was 3.23 (SD 1.26) for the after-school activities. The lowest value was measured in the case of the spare time activity list, similarly to other previous studies, and it was 1.01 (SD 0.12). The Hungarian children selected only a few types of activities from the list on the questionnaire [28,30]. 

The internal consistency of the questionnaire, as measured by the Cronbach alpha, was acceptable, similar to the other studies’ results. According to the criteria applied by George and Mallery, the Cronbach value was acceptable above 0.70, at most 0.80 [47]. In previous studies, the test-retest reliability of the questionnaire was measured between 0.70 and 0.85 (Table 3) [22,30,31,32]. 

Examining the factor structure of the questionnaire based on previous studies’, the uni- and bifactorial structures were tested by confirmatory factor analysis. Similar to Sirajudeen et al.’s study, our findings proved the unifactorial model of the measurement tool to have an acceptable fit [33]. 

The concurrent validity of the PAQ-C questionnaire was tested in association with the body composition data, where weak correlations were found between body composition and the questionnaire. The findings proved that more active children had significantly higher skeletal muscle indexes and lower body fat and body fat % indexes. We found similar results to Isa et al., where body mass index (r = 0.09) and body fat (r = 0.19) were significantly correlated with PAQ-C score [32]. 

The discriminant validity proved gender differences, where boys showed significantly higher activity scores than girls (*p* < 0.001). The average PAQ-C score for girls was 2.87 (SD 1.07), and for boys it was 3.00 (SD 1.05). Our results showed similar patterns to the study by Gobbi et al. (Table 7) [26]. 

Measuring children’s physical activity is essential for several reasons. It allows for the monitoring of overall health and well-being by assessing if the children meet recommended activity guidelines and maintain an active lifestyle. 

The Global Matrix 3.0 highlighted the need for increased efforts to promote physical activity in European children and identified areas for improvement in policies and initiatives related to physical activity promotion [11]. Based on the Global Matrix 4 results, 50% of the Hungarian children spent enough time doing outdoor activities, and the screen time recommendation was not more than 2 h a day, which was fulfilled by only 28.9% of children on weekdays and 32.7% at weekends [48].

Lang et al. explained in their study the importance of physical fitness measurements in children’s monitoring. The importance of monitoring and surveillance methods for children’s physical fitness is essential in a longitudinal way, especially because information is a baseline parameter for decision-making procedures and regarding monitoring, the use of valid and reliable measurement tools is needed [49]. Ács et al.’s studies also aimed at developing the monitoring of PA with validation of the most important PA subjective measurement tools for the Hungarian adult population [16,17]. The Hungarian NETFIT system was developed a decade ago to monitor the physical fitness of the children using different fitness tests. Furthermore, an important fact in students activity patterns was that, since 2012, an every-day physical education method has been applied in all primary and secondary schools in Hungary. All children must be involved daily during physical education classes [50]. 

Additionally, measuring physical activity helps inform the development of interventions and policies aimed at promoting physical activity among children. It provides valuable data for evaluating the effectiveness of programs and interventions, guiding improvements for future initiatives. 

In the cross-sectional study by Pogrmilovic et al., the researchers aimed to assess the availability, comprehensiveness, implementation, and effectiveness of national physical activity (PA) and sedentary behaviour (SB) policies worldwide. Data were collected from 76 countries, with a response rate of 44%. The findings revealed that 92% of the countries had formal written policies for PA, while 62% had policies for SB. Additionally, 62% of countries had national PA guidelines, while 40% had SB guidelines. Only 52% of countries had quantifiable national targets for PA, and 11% had targets for SB [51]. The ministries/departments most involved in promoting PA and reducing SB were those related to sports, health, education, and recreation/leisure. The comprehensiveness of PA policies received a median score of four out of ten, while SB policies scored two. The implementation score for PA and SB policies was six, while the effectiveness score was four for PA and three for SB. Overall, PA policies were better developed and implemented in high-income countries, European countries, and countries in the Western Pacific region compared to low- and lower-middle-income countries. The study concludes that there is a need for increased investment in developing and implementing comprehensive and effective PA and SB policies, particularly in low- and lower-middle-income countries.

The measurement also helps us understand the patterns, contexts, and influences of children’s activity behaviours, identifying factors that promote or hinder their participation in physical activity. Furthermore, accurate measurement contributes to scientific knowledge and research in the field, enabling comparisons across studies and a deeper understanding of the relationship between physical activity and various health outcomes in children. The PAQ-C questionnaire could be a useful part of research examining the health behaviour of children to assess the association between health status and health determinants such as physical activity and to understand the physical activity patterns of schoolchildren for not only researchers but also for physical education teachers to improve physical fitness and motivate students to follow an active lifestyle. Overall, measuring children’s physical activity is crucial to promoting their health, informing interventions, and advancing scientific knowledge in the field [52].

### Limitations of the Study

The study has several limitations. The PAQ-C questionnaire measures the physical activity patterns but not the time spent physically active. Furthermore, our study did not compare PA patterns with objective measurement tools. An objective measurement of PA was also prepared, but due to the lockdown of the COVID-19 pandemic and further consequences, the measurements with accelerometers were miscarried. Consequently, only Phases 1 and 2 of our study were conducted to measure internal consistency, test-retest reliability, and structural, concurrent, and discriminant validity. The questionnaires were filled out using self-reported methods by children, but with the support of a group of experts. The sample was not based on a random selection. Furthermore, seasonal changes in PA patterns were not tested. 

## 5. Conclusions

The findings of our study were provided by the Hungarian version of the PAQ-C questionnaire, which is appropriate for measuring the physical activity patterns of 7–14-year-old children. The questionnaire could be a useful measurement tool for extensive population studies. The findings showed acceptable internal consistency and test-retest reliability of the confirmatory factor analysis, which showed the best fit for the one-factor model of the PAQ-C questionnaire. The newly adapted and tested measurement tool could support the development of new self-reporting questionnaires to measure children’s physical activity levels and provide more relevant information about the health behaviours of the target group.

## Figures and Tables

**Table 1:** Summary of the main characteristics of the sample (N = 620).

Variables	Items	N	%
Gender	male	338	54.52
	female	282	45.48
Mothers’ education	8 classes	53	8.55
	secondary school, vocational school	165	31.29
	college, university	373	60.16
Fathers’ education	8 classes	25	4.03
	secondary school, vocational school	280	45.16
	college, university	315	50.81
Place of living	capital/county seats	446	71.94
	city	76	12.26
	village	98	15.81

**Table 2:** Participant’s body composition results (N = 620).

	Male (N = 338)		Female (N = 282)		Total (N = 620)		Gender Differences
	Mean	SD	Mean	SD	Mean	SD	*p*
Weight (kg)	43.19	15.29	41.79	15.01	42.55	15.17	0.240
Body Fat (kg)	8.18	6.56	9.48	6.27	8.77	6.46	<0.001
Skeletal Muscle Mass (kg)	18.72	6.94	17.06	6.01	17.97	6.58	0.005
Body Mass Index (kg/m^2^)	18.56	3.78	18.34	3.64	18.46	3.71	0.503
Body Fat %	17.71	9.58	21.06	8.04	19.24	9.06	<0.001

**Table 3:** Illustrating the descriptive analysis of the PAQ-C questionnaire.

	Male		Female		Total	
	Mean	SD	Mean	SD	Mean	SD
1. Spare time activity	1.01	0.12	1.02	0.13	1.01	0.13
2. Physical education	4.17	0.99	4.17	0.83	4.17	0.92
3. Recess	2.03	1.08	1.69	0.86	1.88	1.00
4. Lunch	1.04	0.26	1.03	0.28	1.04	0.27
5. After school	3.23	1.26	2.96	1.31	3.11	1.29
6. Evenings	1.78	1.13	1.73	1.20	1.75	1.16
7. Weekends	1.85	0.90	1.78	0.91	1.82	0.91
8. Describes you best	2.85	1.01	2.63	1.04	2.75	1.03
9. Activity frequency	3.11	0.98	2.86	1.02	3.00	1.00

**Table 4:** Internal consistency and test-retest reliability of the PAQ-C questionnaire.

Study	Internal Consistency (Cronbach Alpha)	Test-Retest Reliability (ICC, 95% CI)
Phase I (N = 620)	0.729	0.772 (0.373–0.841)
Phase II (N = 20)	0.736	

**Table 5:** Structural validity fit indexes of the uni- and bivariate structures of the PAQ-C.

PAQ-C	CFI	GFI	TLI	RMSEA	χ^2^	df	*p*	χ^2^/df
Model 1	0.921	0.957	0.891	0.077	120.56	26	<0.001	4.637
Model 2	0.922	0.957	0.896	0.075	120.61	27	<0.001	4.467
Model 3	0.973	0.980	0.961	0.046	57.79	25	<0.001	2.311

**Table 6:** Concurrent validity of PAQ-C total score and body composition indexes by gender (N = 620).

	Male (N = 338)		Female (N = 282		Total (N = 620)	
	r	*p*	r	*p*	r	*p*
Body Fat (kg)	−0.150	0.006	0.029	0.632	−0.084	0.036
Skeletal Muscle Mass (kg)	0.262	<0.001	0.289	<0.001	0.281	<0.001
Body Mass Index	−0.027	0.627	0.075	0.206	0.026	0.520
Body Fat %	−0.275	<0.001	−0.135	0.023	−0.225	<0.001

**Table 7:** Summary table of previous studies’ findings about the PAQ-C questionnaire validation procedure.

Authors	Sample Size	Internal Consistency	ICC	Discriminant Validity—Gender Differences
Italian [26]	1116	0.74	-	Boys significantly higher than girls *p* < 0.001
tCanada [31]	84—congenital heart disease	0.837	0.73	-
Japanese [32]	210	0.80	0.83	Significantly higher in active subgroup *p* < 0.01
Turkish [30]	784	0.77	0.91	-
Chech [22]	Phase 1 = 169 Phase 2 = 63	0.77	0.73–0.94	-

## Data Availability

The data presented in this study are available on request from the corresponding author. The data is not publicly available due to privacy concerns.
